# Effect of transcutaneous electrical acupoint stimulation on remifentanil dosage during craniotomy aneurysm clipping: a prospective, randomized controlled study

**DOI:** 10.1186/s12906-023-04297-x

**Published:** 2023-12-13

**Authors:** Bingyu Wang, Guanfa Peng, Li Chen, Mingling Guo, Jianshun Zhou, Yingying Liu, Zhen Chen, Lifeng Wang

**Affiliations:** 1 The First Clinical College of Medicine, Gannan Medical University, Ganzhou, 34100 China; 2 The Second Hospital of Ningbo, Ningbo, 315100 China; 3 Department of Anesthesiology, The First Affiliated Hospital of Gannan Medical University, No. 128, Jinling West Road, Ganzhou, 34100 China

**Keywords:** Transcutaneous electrical acupoint stimulation, Craniotomy Aneurysm clipping, Remifentanil, Oxidative stress, Brain injury

## Abstract

**Background:**

Craniotomy aneurysm clipping is one of the main treatments for intracranial aneurysm (IA). Endotracheal intubation and intraoperative operation may induce dramatic hemodynamic fluctuations and increase the risk of aneurysm rupture. Intraoperative high-dose opioid use is the main measure to reduce the intraoperative stress response, but it increases the incidence of complications such as postoperative vomiting and delayed awakening. Transcutaneous electrical acupoint stimulation (TEAS) stimulates β-endorphin expression levels and reduces opioid requirements. In this study, we aimed to assess the effects of TEAS on remifentanil dosage and oxidative stress (OS) in craniotomy aneurysm clipping.

**Method:**

Forty-two patients with craniotomy aneurysm clipping were randomized into two groups: the TEAS group (T group) and the sham TEAS group (S group). “Hegu” (LI4), “Neiguan” (PC6) and “Zusanli” points (ST36) were selected, and a “HANS” percutaneous acupoint electrical stimulator was used for intervention 30 min before anesthesia induction until the end of the operation. The primary outcome was intraoperative remifentanil dosage. The secondary outcomes were intraoperative propofol dosage, mean arterial pressure (MAP) and heart rate (HR) 5 min before the TEAS intervention (T_0_), 5 min before head holder pinning (T_1_), immediately after pinning (T_2_), 5 min before craniotomy (T_3_), immediately after craniotomy (T_4_), at craniotomy (T_5_), and at the end of surgery (T_6_), as well as serum β-endorphin levels at T_1_, T_2_ and T_6_ and neuron-specific enolase (NSE), S100β, superoxide dismutase (SOD) and malondialdehyde (MDA) levels at T_1_, T_2_ and 24 h after surgery (T_7_).

**Results:**

The dosage of remifentanil in the T group was reduced compared to that in the S group (*P* < 0.05). At T_2_, T_4_ and T_5_, the MAP and HR in the T group were lower than those in the S group (*P* < 0.05). At T_2_ and T_7_, the levels of NSE, S100β and MDA in group T were lower than those in group S (*P* < 0.05), while the SOD levels in group T were higher than those in group S (*P* < 0.05).

**Conclusions:**

The use of TEAS can reduce the dosage of remifentanil and reduce hemodynamic fluctuations during craniotomy aneurysm clipping. It reduces the occurrence of OS and central nervous system damage during surgery and has a certain brain protective effect.

**Trial registration:**

ChiCTR2100052353. https://www.chictr.org.cn/about.html.

**Supplementary Information:**

The online version contains supplementary material available at 10.1186/s12906-023-04297-x.

## Introduction

Intracranial aneurysm (IA), a cerebrovascular disease with high mortality and disability rates, is a tumor-like protrusion formed by abnormal expansion of the outer and media membranes on the intracranial vascular wall caused by congenital growth and development abnormalities and acquired factors. It is also the major cause of subarachnoid hemorrhage (SAH) [1, 2]. Studies have shown that the overall prevalence rate of intracranial aneurysms in patients aged 35–75 years is 7.0%, including 5.5% in males and 8.4% in females [3].

Craniotomy aneurysm clipping is one of the main methods for the treatment of IA. The key to anesthesia in this surgery is intended to maintain the stability of intraoperative hemodynamics, ensure the stability of the transmural pressure on both sides of the aneurysm (approximately the difference between mean arterial pressure and intracranial pressure), and prevent aneurysm rupture [4]. However, anesthesia induction, tracheal intubation, head holder pinning, craniotomy, tracheal catheter extraction and other operations during surgery can cause drastic fluctuations in hemodynamics, which easily induce aneurysm rupture and bleeding [5]. It is often necessary to increase the dose of opioids during the operation to reduce drastic fluctuations in hemodynamics. However, high doses of opioids may increase the incidence of postoperative complications such as delayed awakening, nausea and vomiting, and postoperative delirium [6–8]. In addition, the central system of patients with craniotomy may be in a state of high oxidative stress (OS) for a long time, and the high oxidative stress of the central nervous system may cause irreversible brain damage after surgery [9, 10]. Therefore, there is an urgent need to find a way to assist analgesia and reduce the central oxidative stress response, which can not only contribute to maintaining the stability of intraoperative hemodynamics but also reduce brain injury.

Transcutaneous electrical acupoint stimulation (TEAS) is a noninvasive and nonpharmacological treatment method based on the meridian theory of traditional Chinese medicine in China and combined with Western transcutaneous electrical nerve stimulation (TENS). Compared with traditional acupuncture techniques, TEAS positioning is simple, requiring only attachment of the gel electrode sheet to the corresponding acupuncture point; low-frequency pulse current is conducted to the acupuncture point through the gel electrode sheet to stimulate the acupuncture point and achieve the purpose of treating diseases and improving prognosis [11]. Studies have shown that TEAS has certain advantages in analgesia [12], OS inhibition and central nervous system protection [13]. Wang et al. performed TEAS intervention for 30 min before anesthesia induction and found that TEAS could reduce the required intraoperative dosage of remifentanil in patients undergoing sinusotomy and reduce the incidence of postoperative dizziness and itching [14]. Yin et al. found that applying TEAS reduced the EC50 of remifentanil in spinal surgery and inhibited the response during extubation [15]. Yuan et al. performed perioperative TEAS intervention in patients with aneurysmal subarachnoid hemorrhage undergoing interventional therapy and found that TEAS may exert a cerebral protective effect by reducing the levels of S100β and neuron-specific enolase (NSE) in serum [16].

With the development of comfort medical care, TEAS is widely used in the clinic. However, the application of TEAS in craniotomy aneurysm clipping is limited. In this study, we aimed to evaluate the effect of applying TEAS on the required intraoperative dosage of remifentanil in patients undergoing selective craniotomy aneurysm clipping, observe whether TEAS can reduce the intraoperative OS, and provide a theoretical basis for the application of TEAS in craniotomy aneurysm clipping.

## Methods

### General information

This is a prospective, double-blind, randomized controlled study that has been approved by the Ethics Committee of the First Affiliated Hospital of Gannan Medical College (LLSC-2,021,101,403) and registered in the Chinese Clinical Trial Registry (ChiCTR2100052353, date of registration: 24/10/2021).

### Participants

Before randomization, all participants were informed of the potential benefits, risks, alternatives, etc., of the study. The patients signed an informed consent form. Participants were selected based on the following criteria.

The inclusion criteria were as follows: (1) age 40–70 years; (2) ASA grade I-III; (3) Hunt grade I-III; and (4) signed clinical informed consent for this study.

Exclusion criteria included the following: (1) refusal to participate in the study; (2) participation in other clinical studies; (3) history of mental illness: due to the influence of various biological, psychological and social environmental factors, the brain is dysfunctional, resulting in cognitive, emotional, volitional and behavioral mental activity disorders; (4) history of alcohol, drug dependence or drug use; (5) history of acupuncture treatment within the past 2 weeks; (6) severe cardiac, liver and renal insufficiency; and (7) patients with potential for medical problems to arise as a result of TEAS, for example, those who have pacemakers or metal implants or are allergic to surface electrodes.

Elimination criteria were as follows: (1) intraoperative bleeding > 800 ml; (2) rupture of intraoperative aneurysm; (3) allergic manifestations such as itching and redness of skin caused by TEAS stimulation; and (4) use of other analgesic drugs or measures before surgery.

### Sample size calculation

In this study, MedCalc 19.0.7 software was used to calculate sample size. According to previous findings, TEAS-assisted general anesthesia for endotracheal intubation could reduce the amount of intraoperative remifentanil by approximately 30% [17], with a type I error of 0.05 and power of 80%. Considering the 20% loss or refusal to visit, 21 patients in each group were required to detect statistical significance, and a total of 42 patients were enrolled in our study.

### Randomization and blinding

SPSS 25.0 software was used to randomize groups. The study subjects were numbered one by one according to the order in which they were enrolled, with the time of the start of the experiment 202,110 (October 2021) as the random number seed. Random numbers are generated using Rv. Uniform function in the SPSS random number generator and then grouped using the SPSS Visual Binning feature. Since this study was a controlled trial randomized into 2 groups, the number of split points was 1, and then 42 patients were randomly divided into Group 1 and Group 2.

Regarding blinding, each subject’s grouping result was written in an opaque and sealed envelope, with the subject’s chronological inclusion number (1–42) on the outside of the envelope, and given to the TEAS operator. This random process was completed by a person who was not involved in the study. After the patient entered the operating room, TEAS was performed by the TEAS operator according to the contents of the envelope. If the envelope contained “1,” the placebo-type Han’s acupoint nerve stimulator was used, and if the envelope contained “2,” the therapeutic Han’s acupoint nerve stimulator was used. TEAS operators knew the contents of the envelopes but did not know the significance of the numbers; the TEAS operators were independent of this study and only administered the TEAS treatment. There was no communication about this study with patients, surgeons, anesthesiologists, or data analysts.

### Anesthesia management

All patients were anesthetized by the same anesthesiologist. After a patient entered the operating room, intravenous access was established and a multifunctional monitor was connected for electrocardiography (ECG) and noninvasive measurement of blood pressure (BP) as well as blood oxygen saturation (SpO2); then, a bispectral index (BIS) monitor (186 − 0106, Kehui Medical Equipment International Trading Co., LTD) was connected. After 30 min of TEAS intervention, general anesthesia was performed for endotracheal intubation; the induction drugs were midazolam 0.05 mg/kg, sufentanil 0.5 µg/kg, propofol 2 mg/kg, and rocuronium 0.6 mg/kg. After endotracheal intubation, mechanical ventilation parameters were adjusted as follows: tidal volume 6–8 ml/kg, respiratory rate 12–16 breaths/minute, inspiratory expiratory ratio 1:2, oxygen flow rate 2 L/min to maintain end-expiratory partial carbon dioxide (P_ET_CO2) at 30–45 mmHg. Then, ultrasound-guided catheterization of the radial artery and the right internal jugular vein on the surgical side was performed to facilitate invasive arterial blood pressure monitoring and blood gas analysis.

Propofol (4–12 mg·kg^− 1^·h^− 1^) and remifentanil (0.05–0.2 µg·kg^− 1^·min^− 1^) were injected intravenously during the operation to maintain the depth of anesthesia, and the injection rate of propofol and remifentanil was adjusted according to hemodynamics and BIS, so that MAP and HR were maintained at the basic values of -20% ~ +20%. BIS was maintained at 40–55. There was intermittent addition of rocuronium to maintain muscle relaxation. Propofol and remifentanil were discontinued at the end of the procedure.

All patients were treated with patient-controlled intravenous analgesia (PCIA) after surgery. The PCIA configuration method was as follows: sufentanil 2 µg/kg + ondansetron 8 mg + 0.9% sodium chloride, configured to total 100 ml.

### TEAS intervention

**Fig. 1:**
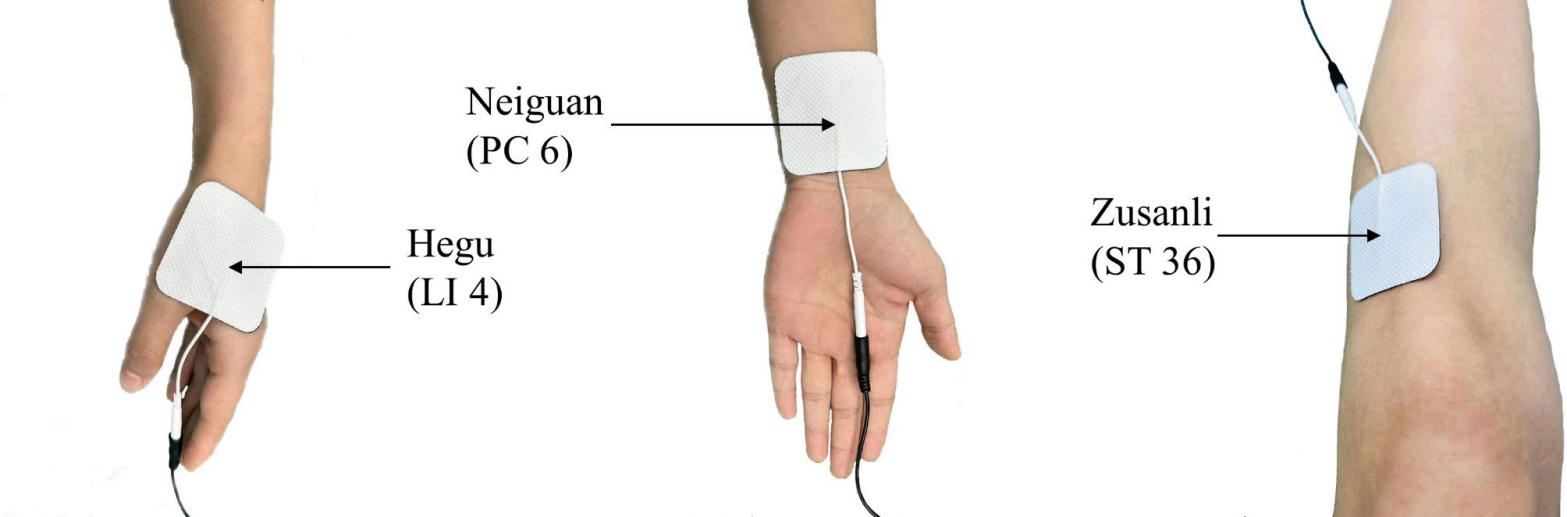
Location of acupoints

The patient’s surgical side to side “Hegu” (LI4, located in the back of the hand, between the first and second metacarpals, the middle point of the radial side of the second metacarpal), “Neiguan” (PC6, located on the anterior aspect of the forearm, 2 inches on the wrist stripes, between the flexor tendon of the radial wrist and the long tendon of the palm) and “Zusanli” (ST36, located on the tibialis anterior muscle, 4 transverse fingers under the external knee, TEAS intervention was performed by opening 1 transverse finger point beside tibial margin) were selected as the acupoints for intervention. Before the gel electrode sheet was pasted on the skin of each patient, the skin surface of the acupoint was wiped clear with clean water, and the skin surface of the acupoint was degreased with 75% alcohol and dried; then, the gel electrode sheet was attached to LI4, PC6 and ST36 with tight pressing. The parameters of the acupoint nerve stimulator were set as follows: time, 30 min before anesthesia induction until the end of surgery; frequency, 2/100 Hz; intensity, < 10 mA (if the stimulation intensity was too high, the patient felt uncomfortable, and the stimulation intensity was reduced and gradually adjusted to the maximum intensity that the patient could tolerate). The placebo-type Han’s acupoint nerve stimulator (Sham TEAS) was used in the Sham TEAS group, while the therapeutic Han’s acupoint nerve stimulator (HANS-200 A, Nanjing Jisheng Medical Technology Co., LTD.) was used in the TEAS group. The two instruments had the same appearance and could both cause numbness in acupoints, but the placebo-type Han’s acupoint nerve stimulator had no therapeutic effect [18] (Fig. 1).

#### Data collection

The general information (sex, age, height, weight, ASA grading) and intraoperative information (operation time, infusion volume, blood loss, urine volume) of all patients were recorded.

#### Primary outcome

Perioperative dosage of remifentanil.

### Secondary outcomes

Perioperative dosage of propofol.

Mean arterial pressure (MAP) and heart rate (HR) 5 min before the TEAS intervention (T_0_), 5 min before head holder pinning (T_1_), immediately after pinning (T_2_), 5 min before craniotomy (T_3_), immediately after craniotomy (T_4_), at craniotomy (T_5_, when the scalp and periosteum separate) and at the end of surgery (T_6_).

Peripheral blood samples of patients at T_0_, T_2_, T_6_ and 24 h after surgery (T_7_) were collected and centrifuged at 3000 g for 10 min, and serum was taken and stored in a -80℃ refrigerator. A commercially available enzyme-linked immunosorbent assay (ELISA) kit (Andygene, USA) was used to detect serum levels of β-endorphins at T_0_, T_2_, and T_6_ and S100β (normal value: 0.068 ~ 0.728 µg·L^− 1^) and NSE (normal value: 0-16.3 ng·ml^− 1^) levels at T_0_, T_2_, and T_7_. The hydroxylamine experimental kit (A001-1, Nanjing Jiancheng Biological Co., Ltd.) was used to measure the level of superoxide dismutase (SOD, normal value: 104 ± 18.8 U·ml^− 1^) in serum at T_0_, T_2_ and T_7_, and a thiobarbituric acid (TBA) experimental kit (A003-1, Nanjing Jiancheng Biological Co., Ltd.) was used to determine malondialdehyde (MDA, normal value: male 4.21 ± 0.57 nmol·ml^− 1^, female 3.99 ± 0.47 nmol·ml^− 1^) levels in serum at T_0_, T_2_ and T_7_. All analyses were repeated and quantified according to the manufacturer’s scheme and averaged for analysis. All serum samples were processed by the same experimentalists in the same laboratory every 3 months.

### Statistical analysis

SPSS 25.0 data analysis software was used to analyze and process the data. The measurement data conforming to the normal distribution are expressed as the mean ± standard deviation (SD), and an independent sample t test was used for intergroup comparisons. Nonnormally distributed measurement data are represented by the median (M) and interquartile range (IQR), and Wilcoxon rank sum tests were used for between-group comparisons. For counting data, we used the number of patients, and the chi-square test or Fisher’s exact test was used for comparisons between groups. For comparison between groups of hemodynamic indicators (MAP, HR) and serological indicators (β-endorphin, SOD, MDA, NSE, S100β) at different time points, repeated measure ANOVA was used, followed by multiple adjustment with Bonferroni correction. A *P*-value < 0.05 was considered statistically significant.

## Results

### Patient characteristics

**Fig. 2:**
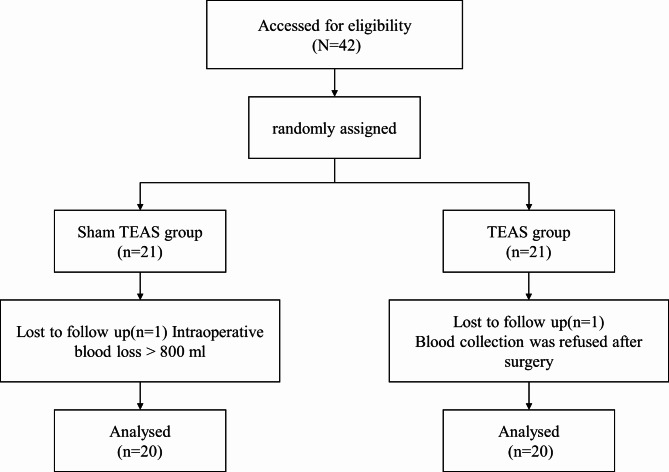
Flow chart

Among the 42 patients, 2 were excluded: One patient in group S withdrew from the study due to massive intraoperative bleeding (blood loss > 800 ml), and one patient in group T withdrew from the study due to the loss of follow-up involving refusal to collect blood samples after surgery. Finally, all the data of 40 subjects were analyzed, with 20 patients in each group (Fig. 2).

The sex [female, 15 (37.5%)], age (56.85 ± 7.73), BMI (22.97 ± 3.56) and ASA grade [ASAII, 40 (20%)] between the 2 groups were similar (*P* > 0.05). Differences in the intraoperative conditions of patients in the two groups, urine volume [1650.00 (1450.00, 2200.00)], operative time (417.05 ± 126.35), infusion volume (3532.5 ± 707.71) and blood loss [450.00 (400.00, 600.00)] were not statistically significant (*P* > 0.05) (Table 1).

**Table 1:** Comparison of general data and intraoperative variables between the two groups

characteristics	Sham TEAS group	TEAS group	*P-*value
Sex(male/female)	10/10	5/15	0.102
Age(year)	58.15 ± 8.54	55.55 ± 7.02	0.299
BMI(kg/m^2^)	22.26 ± 3.21	23.69 ± 3.91	0.216
ASA grading (II/III)	3/17	5/15	0.435
Urine volume(ml)	1700.00(1275.00, 2200.00)	1650.00(1500.00, 2200.00)	0.946
Operation time(min)	404.65 ± 97.26	429.45 ± 154.35	0.547
Infusion volume (ml)	3468.75 ± 553.87	3596.25 ± 859.71	0.580
Blood loss (ml)	400.00(400.00, 525.00)	500.00(300.00, 612.50)	0.546

Data presented as mean ± SD or M and IQR or number of patients. SD- standard deviation; M- median; IQR- interquartile range; BMI- body mass index; ASA- American Society of Anaesthesiologists

### Primary outcome

Compared with group S, the perioperative amount of remifentanil was significantly reduced in group T (*P* < 0.05) (Table 2).

**Table 2:** Intraoperative dose comparison of remifentanil and propofol between the two groups

	Sham TEAS group	TEAS group	*P-*value
Consumption of remifentanil(mg)	2.07 ± 0.63	1.66 ± 0.56^*^	0.036
Consumption of propofol(mg)	1702.00 ± 483.51	1630.00 ± 308.56	0.578

Data presented as mean ± SD. ^***∗***^*P*<0.05 vs. Sham TEAS group. SD- standard deviation

### Secondary outcomes

There was no significant difference in the dosage of propofol between the two groups (*P* > 0.05) (Table 2).

**Fig. 3:**
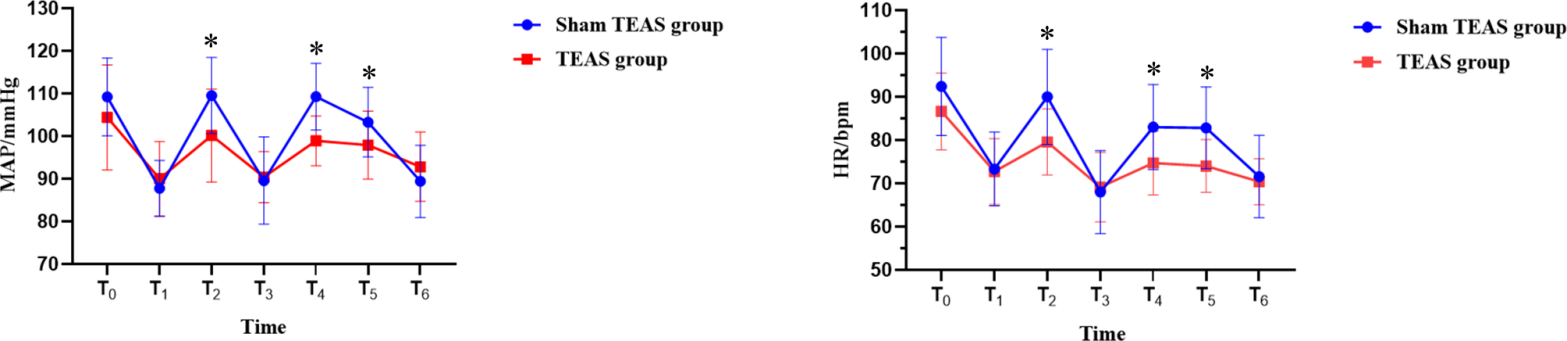
MAP and HR of two groups of patients at distinct junctures Note: Data are presented as the mean ± SD.^*∗*^*P* < 0.05 vs. Sham TEAS group at the same time point

At T_0_, there was no significant difference in MAP and HR between the two groups (*P* > 0.05). At T_2_, T_4_ and T_5_, MAP and HR in group T were lower than those in group S, and the difference was statistically significant (*P* < 0.05) (Table 3; Fig. 3).

**Table 3:** A comparison of MAP and HR at distinct junctures between the two groups

	Group		*P-*value
	Sham TEAS group	TEAS group	
MAP/mmHg			
T_0_	109.20 ± 9.12	104.40 ± 12.32	0.169
T_1_	87.75 ± 6.59	90.00 ± 8.72	0.363
T_2_	109.55 ± 8.94	100.15 ± 10.85^*^	0.005
T_3_	89.60 ± 10.24	90.40 ± 5.97	0.764
T_4_	109.25 ± 7.85	98.90 ± 5.83^*^	0.000
T_5_	103.30 ± 8.18	97.90 ± 7.95^*^	0.041
T_6_	89.40 ± 8.46	92.85 ± 8.15	0.197
HR/bpm			
T_0_	92.40 ± 11.34	86.65 ± 8.89	0.082
T_1_	73.35 ± 8.51	72.75 ± 7.62	0.815
T_2_	90.00 ± 10.99	79.60 ± 7.61^*^	0.001
T_3_	68.00 ± 9.56	69.15 ± 8.01	0.682
T_4_	83.05 ± 9.85	74.70 ± 7.39^*^	0.004
T_5_	82.85 ± 9.45	74.05 ± 6.06^*^	0.001
T_6_	71.60 ± 9.55	70.40 ± 5.31	0.626

Data presented as mean ± SD. ^*∗*^*P*<0.05 vs. Sham TEAS group. SD- standard deviation

**Fig. 4:**
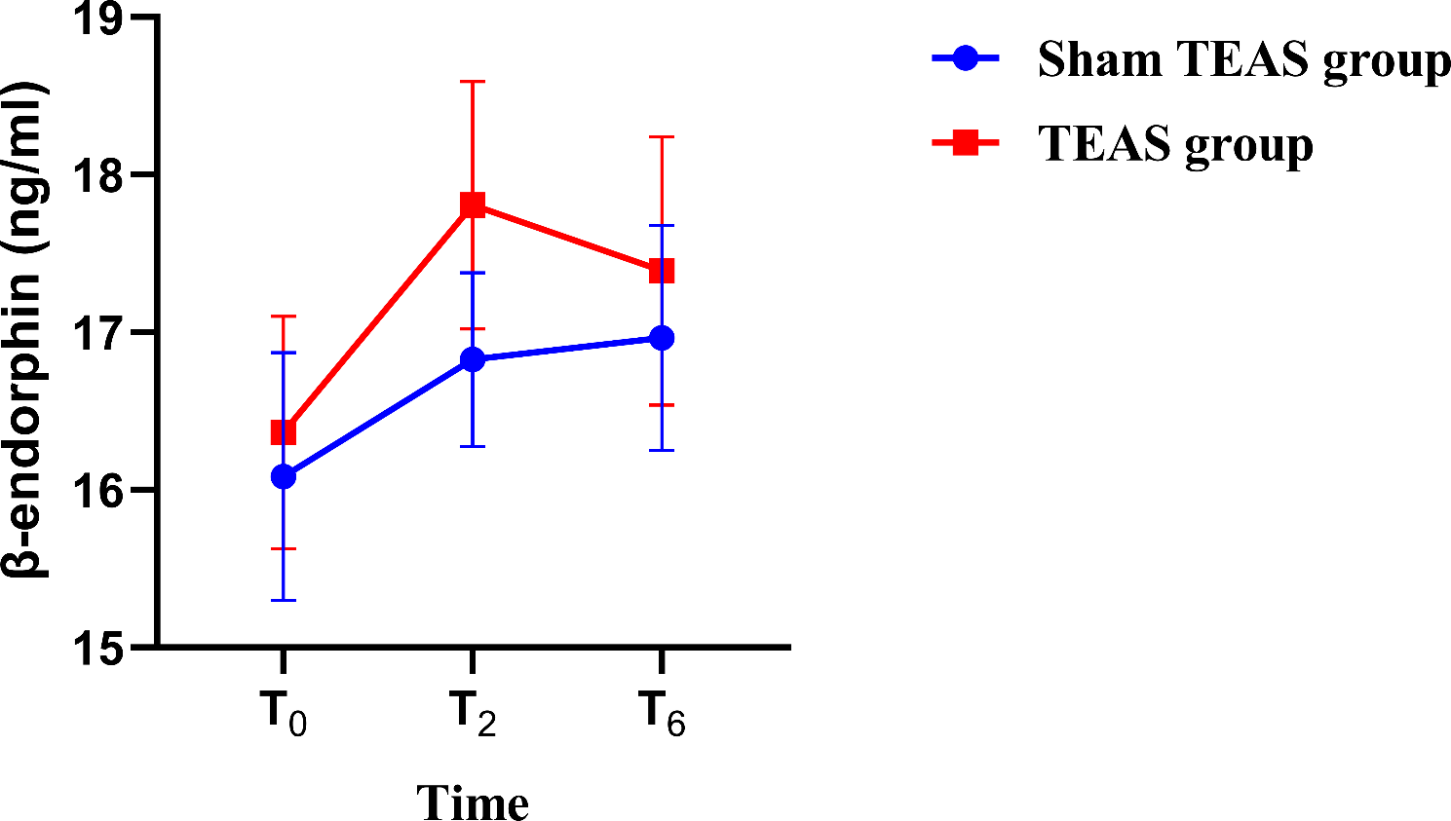
Serum β-endorphin levels in the two groups at distinct junctures Note: Data are presented as the mean ± SE.SE- Standard Error

**Fig. 5:**
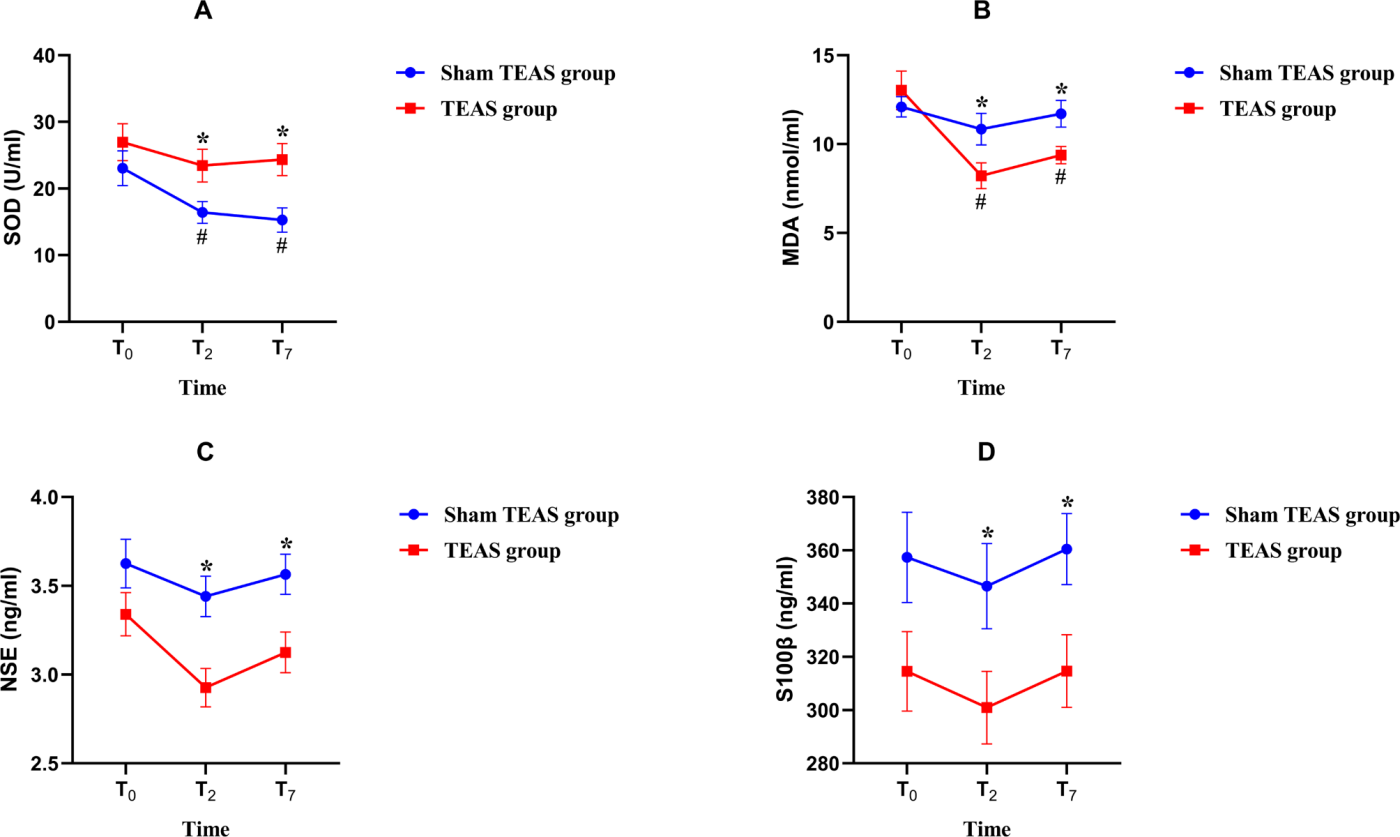
Serum SOD (**A**), MDA (**B**), NSE (**C**) and S100β (**D**) levels at each time point Note: Data are presented as the mean ± SE.^*∗*^*P* < 0.05 vs. Sham TEAS group at the same time point. ^*#*^*P* < 0.05 vs. T_0_ in the same group. SE- Standard Error

There was no significant difference in serum β-endorphin levels at T_0_, T_2_ and T_6_ between the 2 groups (P > 0.05) (Fig. 4). At T_0_, there were no significant differences in the serum levels of NSE, S100β, SOD and MDA between the two groups (*P* > 0.05). Compared with T_0_, SOD levels in group S and MDA levels in group T at T_2_ and T_7_ were decreased (*P* < 0.05). At T_2_ and T_7_, NSE, S100β and MDA in group T were lower than those in group S, while SOD levels were significantly higher than those in group S (*P* < 0.05) (Fig. 5).

## Discussion

IA is a cerebrovascular disease with high risk, high disability rate and high mortality [19]. Clipping craniotomy aneurysm is one of the main treatment methods for IA, but surgical and anesthetic stimulation may cause dramatic circulation fluctuation, induce aneurysm rupture and bleeding, and aggravate postoperative brain injury [20]. In addition, due to the long operation time and great trauma of such surgery, the blocking of cerebral vessels and the pulling or compression of brain tissue during the operation will inevitably cause cerebral ischemia and hypoxia, induce OS and aggravate the degree of brain damage, which is not conducive to the prognosis of patients [21].

As a noninvasive and economical treatment, TEAS meets not only the requirements of the enhanced recovery after surgery (ERAS) concept but also the development of comfort medical care. According to the meridian theory of traditional Chinese medicine, Hegu (LI4), Neiguan (PC6) and Zusanli (ST36) are Yu acupoints and are the main acupoints for analgesia. In studies on the analgesic effects or mechanism of TEAS, many scholars often use these three acupoints compatibly [14, 22]. “Hegu” is used to treat fever, headache and trigeminal neuralgia and has sedative and analgesic effects [23]. “Neiguan” is used for headache and psychonervous system diseases and has analgesic and central nervous protection effects [24, 25]. “Zusanli”, mainly used to treat headache and dizziness, has analgesic effects and enhances body immunity [26]. In traditional Chinese medicine treatment, “Neiguan” and “Zusanli” are commonly used for pain relief. The compatible application has complementary effects and thus improves the pain relief effect. Studies have shown that the compatible application of “Zusanli” and “Neiguan” in TEAS therapy can inhibit the stress response and protect the central nervous system [27, 28]. According to the theory of traditional Chinese medicine, the treatment of headache generally takes “Hegu” in the large intestine channel of Yangming. Finally, “Hegu”, “Neiguan” and “Zusanli” were selected as the stimulation points for this study.

Inhaled anesthetics are effective vasodilators that can be used to maintain the stability of perioperative circulation, but they carry the risk of causing increased intracranial pressure during neurosurgery [29]. Propofol and remifentanil pumps have unique advantages in neurosurgery; they can effectively avoid cerebral vascular dilation caused by inhalation anesthesia, reduce intracranial pressure, and reduce haemodynamic fluctuations during craniotomy and are superior to inhalation anesthesia [30]. Zhou et al. found that remifentanil combined with propofol could better maintain the stability of intraoperative hemodynamic parameters, reduce the incidence of postoperative adverse reactions and improve the prognosis of patients during craniotomy tumor surgery [31]. Therefore, all intravenous anesthesia was also adopted in this study, and intraoperative drugs were injected by an intravenous pump with remifentanil and propofol. The results of this study found that the dosage of propofol in the two groups was similar, but the dosage of remifentanil in the T group was lower in the perioperative period than that in the S group. This is consistent with the findings of Yin et al. [15]. Moreover, MAP and HR in the two groups were significantly increased at T_2_, T_4_ and T_5_, but the values in group S were higher than those in group T, and the difference between the two groups was statistically significant (*P* < 0.05). This suggests that TEAS can reduce the dosage of opioids and maintain hemodynamic stability after intervention. This may be related to inducing the release of endogenous opioid peptides (such as enkephalins, endorphins and dynorphins) at the central level to produce analgesic effects [32]. β-Endorphin is a major analgesic substance released by the pituitary gland that plays an important role in self-analgesia [33]. It can inhibit the release of pain stimulus transmitters such as substance P and block the pain stimulus conduction pathway by binding with µreceptors, thus playing an analgesic role [34]. TEAS intervention has been found to increase serum β-endorphin concentrations when it exerts its analgesic effect and reduces opioid use [35]. Qi et al. found that TEAS intervention during labor analgesia can reduce labor pain, increase serum β-endorphin levels, and shorten the first and second stages of labor [36]. However, in this study, there was no significant difference in serum β-endorphin levels between the two groups at T_2_ and T_6_ (*P* > 0.05). On the one hand, we suspect that this result may be related to prolonged TEAS interventions. Studies have shown that repeated TEAS intervention over a long period of time or at short intervals will cause the body to adapt to TEAS and thus become highly tolerant of analgesia [37]. In this study, TEAS was initiated 30 min before anesthesia induction and lasted until the end of surgery, and the stimulation time was generally approximately 6 h, which was relatively long and thus led to analgesic tolerance. We suspect that the mechanism of analgesic tolerance may be related to continuous TEAS stimulation in that it keeps the β-endorphins concentration at a relatively stable level. On the other hand, endogenous analgesic mediators induced by TEAS may function directly in the central nervous system, but the level of β-endorphin in peripheral blood does not rise, because the increased amounts of these mediators do not affect their peripheral blood concentrations [14].

OS is the basis of cerebrovascular disease development [38]. The brain is sensitive to OS. When the body is subjected to various harmful stimuli, it will promote the inflammatory infiltration of neutrophil granulocytes, increase the secretion of proteases, and produce a large number of highly active molecules, leading to an increase in the concentration of reactive oxygen species/nitrogen (ROS/RNS) and initiating central nervous system damage [39]. SOD and MDA are important markers of oxidative stress. SOD belongs to the enzyme antioxidant system and is an important scavenger against superoxide anions in the body. It can effectively remove free radicals by catalyzing the auto-oxidation‒reduction reaction of superoxide anion free radicals and maintain the dynamic balance of free radicals in the body, which is considered the first line of defense in the antioxidant system [40]. MDA is the end product of lipid peroxidation in cells. It is the unsaturated fatty acid produced by the action of oxygen free radicals on biomembranes, which can reflect the severity of lipid peroxidation in the body and the severity of cell damage caused by free radical attack. Due to its accumulation, it can cause the cross-linking polymerization of nucleic acids, proteins and other macromolecules, resulting in the destruction of the integrity of the cell membrane and then affecting its physiological function and causing cytotoxicity [41]. Studies have shown that the traumatic stimulation of head holder pinning during craniotomy can stimulate the body to produce a strong stress response, induce OS, lead to endothelial cell dysfunction, vascular remodeling and blood-brain barrier injury, and then cause cerebral ischemia reperfusion injury and damage to brain tissue [38, 42]. The application of TEAS can inhibit OS and reduce brain damage by regulating the nuclear factor erythroid-2 related factor 2 (Nrf2)/heme oxygenase-1 (HO-1) signaling pathway [13]. Zhuang et al. found in lower extremity surgery using tourniquets that TEAS intervention 30 min before surgery until the end of surgery could reduce serum SOD and MDA levels and reduce OS [43]. In craniotomy, Ni et al. also found that TEAS may reduce brain damage by reducing lipid peroxidation [44]. This is consistent with the results of this study. This study revealed that at T_2_ and T_7_, the SOD level in the T group was higher than that in the S group, and the MDA level in the T group was lower than that in the S group (P < 0.05). This suggests that the application of TEAS reduces the occurrence of OS during craniotomy aneurysm clipping.

NSE is a specific acid protease of neurons and neuroendocrine cells. S100β is a specific biological protein in the central nervous system, approximately 96% of which exists in the brain and is mainly produced by astrocytes. Under physiological conditions, the concentrations of NSE and S100β in serum are extremely low. After central nervous system injury, the integrity of nerve cells is destroyed, and NSE and S100β are released from cells into the blood, resulting in high expression levels of NSE and S100β in peripheral blood [45]. Studies have shown that NSE and S100β are the signature proteins of central nervous system injury and are crucial for predicting the prognosis of brain injury [46]. Studies have found that TEAS can reduce the levels of NSE and S100β in the serum of surgical patients, and reduce the damage to the central nervous system caused by anesthesia and surgery [28, 47]. Wang et al. also verified this conclusion by conducting a TEAS intervention during craniotomy [48]. At the same time, this study also found that the levels of NSE and S100β in the T group were lower than those in the S group (*P* < 0.05), which was consistent with the study by Wang et al. [48], indicating that TEAS can reduce the incidence of brain injury during craniotomy aneurysm clipping and has a certain central protective effect.

This study has certain limitations. As this was a single-center study, the sample size was small, and further verification of the accuracy of the experimental results will increase the sample size. In addition, in order to objectively reflect the effects of TEAS on analgesic effect, oxidative stress injury and brain injury, only specific factors were selected as observation indicators in this study. More indexes, such as Methionine enkephalin, ROS/RNS, Neurofilament (NF), etc., were not used. Second, since the patients in this study were transferred to the neurosurgical intensive care unit for further treatment and monitoring after surgery, the follow-up of the patients in this study was only conducted 24 h after surgery, and the postoperative recovery of the patients was not followed up for a long time.

## Conclusion

In conclusion, perioperative TEAS intervention can reduce the dosage of remifentanil and maintain hemodynamic stability. At the same time, our study also found that perioperative TEAS intervention can also reduce the occurrence of oxidative stress in patients undergoing craniotomy aneurysm clipping and provide certain brain protection.

### Electronic supplementary material

Below is the link to the electronic supplementary material.

Supplementary Material 1

## Data Availability

All data supporting the results of this study are available from the corresponding authors. Data may be provided to the author upon reasonable request.
