# Supplementary material for: Effect of transcutaneous electrical acupoint stimulation on remifentanil dosage during craniotomy aneurysm clipping: a prospective, randomized controlled study
Source: BMC Complement Med Ther. 2023 Dec 13;23:453. doi: 10.1186/s12906-023-04297-x (PMC10717748; doi:10.1186/s12906-023-04297-x)
Supplement: Supplementary file 1 — Supplementary Material 1 [file 12906_2023_4297_MOESM1_ESM.zip › 12906_2023_4297_ReviewerReport_V1_R1.docx]

**FEEDBACK FOR AUTHOR(S)**

Dear Authors

It is an interesting study showing the effect of TEAS on intraoperative HDs and stress biomarkers and concluded that TEAS intervention reduces the haemodynamic fluctuations, dosage of opioids, and occurrence of oxidative stress in patients undergoing craniotomy aneurysm clipping surgery.

Even though the article is well written, there are a few points that needs to addressed.

1. This article examines the multiple primary outcomes. How did you adjust the multiplicity or reduce familywise error rate? In statical tests adjustment was not applied.

2. Sample size was calculated using the dose of remifentayl required for ETT. It may not be appropriate in reflecting the sample size of all the parameters used in primary outcome. Hence, it is highly underpowered study.

3. Discussion: The first para includes too lengthy introduction to the topic. It should mainly discuss the findings. Discussion should be focused to support and describe the results.

4. Conclusion: As the sample size is quite small, the last line should be omitted and the future studies should be advised with appropriate sample size for better understanding the intraoperative role of TEAS.

**FEEDBACK FOR EDITOR**

Your recommendation

Revise

Is the study design appropriate to answer the research question (including the use of appropriate controls), and are the conclusions supported by the evidence presented?

Yes

Are the methods sufficiently described to allow the study to be repeated?

Yes

Is the use of statistics and treatment of uncertainties appropriate?

No

Comments: The adjustments are made for multiple primary outcomes. Hence, the findings may be by chance rather than true positives.

Is the presentation of the work clear?

Yes

Are the images in this manuscript (including electrophoretic gels and blots) free from apparent manipulation?

Yes

Confidential Comments to the Editor

Respected Editor

The study needs minor corrections as advised in the comments.

Regards
