# Supplementary material for: Effect of transcutaneous electrical acupoint stimulation on remifentanil dosage during craniotomy aneurysm clipping: a prospective, randomized controlled study
Source: BMC Complement Med Ther. 2023 Dec 13;23:453. doi: 10.1186/s12906-023-04297-x (PMC10717748; doi:10.1186/s12906-023-04297-x)
Supplement: Supplementary file 1 — Supplementary Material 1 [file 12906_2023_4297_MOESM1_ESM.zip › 12906_2023_4297_ReviewerReport_V1_R2.docx]

FEEDBACK FOR AUTHOR(S)

The grammar particularly, and the language in general, needs a thorough revision. For example, the sentences in line 55, 61, 73, 125, 222-231, 282... are incomprehensible.

71: It should be noted that remifentanil causes hyperalgesia at high doses.

85: this statement is not entirely true. This study does not demonstrate advantages in analgesia, but rather that it decreases the chronification of pain.

86: this statement is not entirely accurate

116: Please define mental illness

117: Please define "recent" acupuncture treatment

121: Elimitacion criteria is not correct

125:It is not well understood that the sample size has been calculated based on [17]. Please reword it better.

131: "al least 42 participants were included" mixes future and past.

131: Figure 1 goes to results and not to methods.

145: Stimulator manufacturer goes at this point

150: not understandable

155: manufacturers of the monitors?

163: In a study on intracranial surgery, carbon dioxide values, which may have effects on oxidative stress and biochemical markers of brain injury, should be monitored more precisely.

169: "according to hemodynamics": It is not explained in the methodology how remifentanil is titrated.

181: What does Tetras intervention mean?

195: What does the Sham TEAS not do to be able to claim that it has no therapeutic effect?

201: The primary outcome is just one, not a variety of parameters.

202: If remifentanil is titrated according to hemodynamic status, MAP and HR cannot be an outcome of the study, but a variable to be controlled.

205: Please specify what is meant by T5, "at craniotomy".

210: Please explain 3000 x g

221: statistical analysis is not correctly explained

233: How many subjects were eligible for the study?

239: Since it is a randomized study, it is not necessary to perform statistical analysis of the demographic variables, only descriptive.

246: clearly specify when the result of the primary outcome is presented. The dosage of propofol was not an expected outcome in the metrics.

253: Presenting the same results in a table and figure is repetitive.

256: Presenting the same results in a table and figure is repetitive.

262: Presenting the same results in a table and figure is repetitive.

Discusion: Repeatedly, claims are made about the benefits of TEAS based on single studies in rodents and dogs, without explaining that they are not in humans.

362: What does Nrf2/HO-1 mean?

Nowhere in the text is it explained what are the normal laboratory values for all biochemical variables. This is relevant because according to my hospital's laboratory values, the majority of measurements are within normal values.

Table 1: The meaning of all acronyms should be explained

Table 4 -5: The normal limits of the laboratory should be given. In the case of MDA, there are different values of normality according to gender. The meaning of all acronyms should be explained

FEEDBACK FOR EDITOR

Your recommendation

Revise

Is the study design appropriate to answer the research question (including the use of appropriate controls), and are the conclusions supported by the evidence presented?

No, but these points can be addressed with revisions

Comments: The outcomes of the study should be more clearly explained. They cannot use a clinical variable (hemodynamic status) to regulate remifentanil dosing, and then later decide that this same clinical variable is an outcome of the study.

Are the methods sufficiently described to allow the study to be repeated?

No, but these points can be addressed with revisions

Comments: They should explain the effect of the placebo stimulator.

They should explain how they perform the intraoperative hemodynamic management, since this influences the primary outcome.

Since the study talks about intracranial surgery, they should explain how they handle plastmatic carbon dioxide, since hyper- or hypocapnia can have an effect on oxidative stress and biochemical markers of brain damage.

They should give their laboratory's reference values for the biochemical variables they measure.

Is the use of statistics and treatment of uncertainties appropriate?

No

Comments: The statistical study they have conducted is not adequately explained. They need to explain which test they have applied to each type of variable more clearly.

Is the presentation of the work clear?

No, it’s not suitable for publication unless extensively edited

Comments: Grammar needs a deep revision. Occasionally, the vocabulary used does not correspond to that commonly used in medical language.

Are the images in this manuscript (including electrophoretic gels and blots) free from apparent manipulation?

Yes

Confidential Comments to the Editor

Since this is a study about remifentanil, a drug that produces hyperalgesia at high doses (a fact that is not mentioned), it is necessary to collect data about postoperative pain.

They repeatedly make statements about the benefits of TEAS based on single studies in rodents and dogs, without explaining that they are not in humans.

Occasionally they make statements based on a reference that dooes not prove this statement.

They talk about statistically significant differences in biochemical variables without giving the normality value of their laboratory. According to the laboratory at my hospital, the results are always within the normal range. That is, it has no clinical repercussions.

The Chinese clinical trial registry is not open access. I would ask them for a copy of the registry to check if they have changed the objectives.
