# Supplementary material for: Effect of transcutaneous electrical acupoint stimulation on remifentanil dosage during craniotomy aneurysm clipping: a prospective, randomized controlled study
Source: BMC Complement Med Ther. 2023 Dec 13;23:453. doi: 10.1186/s12906-023-04297-x (PMC10717748; doi:10.1186/s12906-023-04297-x)
Supplement: Supplementary file 1 — Supplementary Material 1 [file 12906_2023_4297_MOESM1_ESM.zip › 12906_2023_4297_ReviewerReport_V2_R1.docx]

**FEEDBACK FOR AUTHOR(S)**

The Authors have addressed the comments efficiently.

The manuscript may be accepted.

Regards

**FEEDBACK FOR EDITOR**

Your recommendation

Accept

Is the study design appropriate to answer the research question (including the use of appropriate controls), and are the conclusions supported by the evidence presented?

Yes

Are the methods sufficiently described to allow the study to be repeated?

Yes

Is the use of statistics and treatment of uncertainties appropriate?

Yes

Is the presentation of the work clear?

Yes

Are the images in this manuscript (including electrophoretic gels and blots) free from apparent manipulation?

Yes

Confidential Comments to the Editor

Respected Editor,

The manuscript may be accepted as the comments are satisfactorily addressed.

Regards
